# Supplementary material for: Adaptive immunity shapes baseline physiology of M. tuberculosis in high-dose versus low-dose infection BALB/c mouse drug treatment models
Source: Antimicrob Agents Chemother. 2026 Jul 31;70(9):e00421-26. doi: 10.1128/aac.00421-26 (PMC13712817; doi:10.1128/aac.00421-26)
Supplement: Supplementary file 1 [file aac.00421-26-s0001.docx]

**SUPPLEMENTAL INFORMATION**

**Adaptive immunity shapes baseline physiology of M. tuberculosis in high-dose versus low-dose infection BALB/c mouse drug treatment models**

Jo Hendrix, Reem Al Mubarak, Karen Rossmassler, Holly Nielson, Elizabeth Wynn, Camile Moore, Isabelle L. Jones, Martin I Voskuil, Brendan Podell, Gregory T. Robertson, Chuangqi Wang, Nicholas D. Walter

Table of Contents

[1. Supplemental Methods	3](#_Toc233148482)

[***1.1. RNA extraction***	3](#_Toc233148483)

[***1.2. Library preparation***	3](#_Toc233148484)

[***1.3. Sequencing and Data Preparation***	4](#_Toc233148485)

[***1.4. Clustering of Host Genes***	5](#_Toc233148486)

[***1.5. Clustering of Mtb genes***	7](#_Toc233148487)

[***1.6. Table S1.*** *Curated Mtb gene categories*	10](#_Toc233148488)

[***1.7. Mtb Average Expression Plots***	13](#_Toc233148489)

[2. Supplemental Results	15](#_Toc233148490)

[***2.1 Host gene clustering***	15](#_Toc233148491)

[***2.2 Gene set enrichment of host gene clustering***	16](#_Toc233148492)

[***2.3. PCA of Host samples***	17](#_Toc233148493)

[***2.4. Host pair-wise comparison of transcriptome between timepoints***	18](#_Toc233148494)

[***2.5. Expression changes during late infection***	19](#_Toc233148495)

[**2.6. Table S2.** *Expression changes of select host immune genes*	21](#_Toc233148496)

[***2.7. Bacterial gene clustering***	24](#_Toc233148497)

[***2.8. Table S3.*** *Gene categories that were significantly enriched in each gene cluster*	25](#_Toc233148498)

[***2.9. Additional average expression plots of gene categories in LDA model***	26](#_Toc233148499)

[***2.10. Expression changes in Sigma Factor genes***	27](#_Toc233148500)

[***2.11. Expression changes in Toxin genes***	28](#_Toc233148501)

[***2.12. Concordance of host and Mtb transcription***	32](#_Toc233148502)

[***2.13. DGEA in HDA model***	35](#_Toc233148503)

[***2.14. Gene categories enriched in genes differentially expressed between models***	36](#_Toc233148504)

[3. References	38](#_Toc233148505)

# **1. Supplemental Methods**

## ***1.1. RNA extraction***

Murine tissue samples were snap frozen in 7 mL Precellys tubes (Bertin CKMix50.cat# P000939-LYSK0-A) under liquid nitrogen. To each tissue sample, 1.5 mL GTC-TCEP buffer^1^ was added and samples were homogenized at 7,200 rpm for 16s using a Precellys Evolution (Bertin). Homogenates were incubated at room temperature for 5 minutes while protected from light. Unlysed mycobacterial cells were pelleted at 10,000g for 3 minutes, then resuspended in 0.8 mL RLT+ BME buffer (Qiagen, TCEP concentration 0.75 mM). Mycobacterial cells were lysed in 2 mL Precellys tubes (Bertin CK01. cat # P000919-LYSK0-A) as above using a Precellys Evolution (Bertin) at 6,500 rpm for 30s three times, resting on ice for 5 minutes each time, and stored at -80⁰C.

After storage at -80⁰C, murine lysates were thawed, and RNA was purified using the Maxwell simplyRNA tissue kit (murine samples) using the Maxwell RSC instrument following the manufacturer’s instructions with the following modifications. Additional kit DNase was added at twice the manufacturer’s recommended amount. Purified RNA was quantified using the Quantifluor RNA system (Promega).

## ***1.2. Library preparation***

Samples were prepared for SEARCH-TB using Illumina’s AmpliSeq for Illumina Custom and Community RNA Panels kit, according to the Illumina’s recommendations except where noted. RNA was diluted to a concentration of 15-30 ng/uL, and reverse transcribed. cDNA targets were amplified with the SEARCH-TB panel with the following thermocycling conditions: 99°C for 2 minutes, 18 cycles of 99°C for 15 seconds and 60°C for 8 minutes, hold at 10°C. Amplicons were partially digested, indexes were ligated, and the library was cleaned. The library was amplified, with the following thermocycling conditions: 98°C for 2 minutes, 9 cycles of 98°C for 15 seconds and 64°C for 1 minute, hold at 10°C. The library was cleaned a second time, quantified via Qubit using the dsDNA kit (Invitrogen), and evaluated for quality via TapeStation with the High Sensitivity D1000 ScreenTape (Agilent). Libraries were diluted to the same concentration and checked for intact sequencing adaptors via qPCR. Inserts were 124-139 bp, yielding amplicons of approximately 280 bp.

## ***1.3. Sequencing and Data Preparation***

Libraries were sequenced on an Illumina NovaSeq6000 at the University of Colorado Anschutz Medical Campus Genomics Shared Resource, 2x150. Bioinformatics analyses were performed on the Alpine Server at the University of Colorado Anschutz Medical Campus.

## ***1.4. Clustering of Host Genes***

Host genes (29,579 in total) were filtered to retain the most variable genes. To determine this threshold, the standard deviation was first calculated across all samples for each gene. Next, the average and standard deviation of these values were calculated. The average standard deviation was 0.680463817615267 with a standard deviation of 0.223007625516162. Finally, the threshold was calculated as this overall average– 0.5*standard deviation = 0.680463817615267 – 0.5*0.223007625516162 = 0.568960004857186. Genes with a variance greater than this threshold were retained, resulting in 18,354 genes, for clustering.

Auto-correlation coefficients (ACF) were then computed for each gene to capture temporal dynamics across all time points. Genes were then clustered via community clustering based on Euclidean distance.

A variety of K values were tested for community clustering. We tested values from 3000 to 5000 in increments of 200. The final K value of 4000 was chosen based on picking the K value that resulted in the highest Silhouette value (**Fig S1**).

**Fig S1.** Silhouette plot of hierarchical clustering of 18,354 mouse genes using a K value of 4000.

Since ACF captures temporal dynamics but not directionality, the resulting clusters contained symmetric patterns. To separate them, we split clusters based on whether gene expression at the first time point was greater than zero which resulted in eight clusters for the host genes.

## ***1.5. Clustering of Mtb genes***

*Mtb* genes (3,568 in total) were filtered to retain the most variable genes. To determine this threshold, the standard deviation was first calculated across all samples for each gene. Next, the average and standard deviation of these values were calculated. The average standard deviation was 0.751272710943465 with a standard deviation of 0.261479483151631. Finally, the threshold was calculated as this overall average– 1.5*standard deviation = 0.751272710943465 – 1.5*0.261479483151631= 0.359053486216018. Genes with a variance greater than this threshold were retained, resulting in 3,317 genes, for clustering.

Auto-correlation coefficients (ACF) were then computed for each gene to capture temporal dynamics across all time points. Genes were then clustered via community clustering based on Euclidean distance.

A variety of K values were tested for community clustering. We tested values from 200 to 650 in increments of 50. After finding higher Silhouette values around K = 550, we tested values between 500 and 600 in increments of 10. The final K value of 540 was chosen based on picking the K values that resulted in the highest Silhouette value (**Fig S2**).

**Fig S2**. Silhouette plot of hierarchical clustering of 3,317 *Mtb* genes using a K value of 540.

Since ACF captures temporal dynamics but not directionality, the resulting clusters contained symmetric patterns. To separate them, we split clusters based on whether gene expression at the first time point was greater than zero which resulted in ten clusters for the *Mtb* genes (**Fig S3**). In these split clusters, g1a and g1b are symmetrical. These clusters were then consolidated based on similar temporal expression patterns. Clusters g1a and g2a were combined into Cluster 1. Clusters g3a and g5a were combined into Cluster 3. Cluster g4a and g4b were left as they were and renamed Cluster 2 and Cluster 5, respectively. Clusters g3b and g5b were combined into Cluster 4. Clusters g1b and g2b were combined into cluster 6.

**Fig S3**. Average expression of the ten gene clusters obtained after splitting the original clusters to separate symmetrical patterns.

However, after separating and merging the clusters, there was a small set of genes that demonstrated the pattern of its symmetrical cluster (**Fig S4**). The genes circled in Cluster 1 of **Fig S4** were reassigned to Cluster 6. The genes circled in cluster 6 of **Fig 4S** were reassigned to Cluster 1.

**Figure S4.** Gene expression of all genes per cluster. Each dot represents the average gene expression of a gene across all samples available for a given time point. Lines connect the dots. Blue and pink circles indicate the genes that were reassigned to a different cluster.

## ***1.6. Table S1.*** *Curated Mtb gene categories*

Gene categories curated from the literature were used in category enrichment analysis. Along with the reference, the total number of genes from each source along with the number of genes from each source which were in the SEARCH-TB assay is given. Other gene sets used for enrichment analysis are available in Cole *et al.*, 1998^2^.

| **Category** | **Genes Total** | **Genes In Assay** | **Source** |
| --- | --- | --- | --- |
| ABC transporters | 72 | 68 | ^3^ |
| ABC transporters - Anion Transporters | 7 | 7 | ^4^ |
| ABC transporters - Metal Transporters | 5 | 5 | ^4^ |
| ABC transporters - Type I peptide and amino acids | 8 | 6 | ^4^ |
| ABC transporters - Type I phosphate | 8 | 8 | ^4^ |
| ABC transporters - Type I Sugar Importers | 12 | 12 | ^4^ |
| Alternative ribosomal proteins | 5 | 4 | ^5^ |
| Antigen 85 | 3 | 3 | ^6^ |
| Antitoxins | 76 | 72 | ^7^ |
| Arabinogalactan (AG) | 19 | 18 | ^8^ |
| Beta Oxidation | 18 | 18 | ^9^ |
| Cell wall synthesis | 40 | 40 | ^10^ |
| Cholesterol A and B ring degradation | 10 | 10 | ^11^ |
| Cholesterol C and D ring degradation | 5 | 5 | ^11^ |
| Cholesterol side chain degradation | 33 | 33 | ^11^ |
| Cutinase-Like Protein (CULP) | 7 | 7 | ^12^ |
| Cytochrome *bcc/aa3* supercomplex | 6 | 6 | ^13^ |
| Cytochrome *bd* oxidase | 3 | 3 | ^13^ |
| Diacyltrehalose (DAT) and Pentaacyltrehalose (PAT) | 5 | 5 | ^14^ |
| DNA replication and repair | 27 | 25 | ^15^ |
| DosR | 48 | 48 | ^16^ |
| Efflux Pumps and Transports | 26 | 25 | ^17^ |
| Enduring Hypoxic Response | 161 | 149 | ^18^ |
| Esterases (Lip family) | 22 | 20 | ^12^ |
| ESX1 | 19 | 18 | ^19^ |
| ESX2 | 12 | 12 | ^19^ |
| ESX3 | 11 | 9 | ^19^ |
| ESX4 | 7 | 7 | ^19^ |
| ESX5 | 15 | 11 | ^19^ |
| Fatty Acid Synthases I | 1 | 1 | ^2^ |
| Fatty Acid Synthases II | 9 | 8 | ^20^ |
| Fumarate reductase | 4 | 4 | ^2^ |
| Kas operon | 5 | 3 | ^21^ |
| kstR1 regulon | 71 | 70 | ^22^ |
| kstR2 regulon | 15 | 14 | ^22^ |
| LAM | 15 | 14 | ^23^ |
| LpqY-SugA-SugB-Sug trehalose transporter | 5 | 5 | ^4^ |
| Mce1 | 7 | 7 | ^2^ |
| Mce2 | 7 | 6 | ^2^ |
| Mce3 | 7 | 7 | ^2^ |
| Mce4 | 7 | 7 | ^2^ |
| mmpL | 14 | 14 | ^24^ |
| mmpS | 5 | 5 | ^25^ |
| Mycobactin Biogenesis | 10 | 10 | ^26^ |
| Mycolic Acid Modification | 12 | 12 | ^27^ |
| Mycolic Acid Transfer and Modification | 7 | 7 | ^27^ |
| NADH dehydrogenase type I | 14 | 12 | ^28^ |
| NADH dehydrogenase type II | 2 | 2 | ^28^ |
| Nitrate import and reductase | 8 | 6 | ^2^ |
| Oxidative Stress | 49 | 48 | ^29^ |
| Phthiocerol Dimycocerosate (PDIM) | 20 | 20 | ^30^ |
| Peptidoglycan (PG) | 34 | 32 | ^31^ |
| PG synthesis1- cytoplasmic steps | 7 | 7 | ^31^ |
| PG synthesis2- membrane-associated steps | 3 | 3 | ^31^ |
| Peptidoglycan (PG) Linking | 13 | 13 | ^31^ |
| PG synthesis4- remodeling and degradation | 2 | 2 | ^31^ |
| Peptidoglycan (PG) Transport and recycling | 3 | 3 | ^31^ |
| Phospholipases C | 4 | 4 | ^32^ |
| Primary Ribosomal Protein | 53 | 50 | ^2^ |
| response to acid stress | 11 | 9 | ^33^ |
| Ribosomal hibernation | 4 | 4 | ^34^ |
| Sigma Factors | 13 | 12 | ^3^ |
| Stringent Response - Induced | 70 | 58 | ^35^ |
| Stringent Response - Repressed | 78 | 66 | ^35^ |
| Succinate Dehydrogenase Types I and II | 7 | 7 | ^36^ |
| Toxin-Antitoxin | 152 | 146 | ^7^ |
| Toxins | 76 | 74 | ^7^ |
| Transcription Factors | 198 | 187 | ^3^ |
| Trehalose | 10 | 10 | ^37^ |
| Triacylglycerol Synthases | 16 | 14 | ^38^ |
| UgpABCE glycerophosphocholine transporter | 4 | 4 | ^4^ |
| Universal Stress Proteins | 10 | 9 | ^3^ |
| WhiB-Like transcription factors | 7 | 7 | ^39^ |
| Zur regulon | 20 | 17 | ^40^ |

* Primary Ribosomal Proteins include ribosomal proteins identified by Cole *et al.* but exclude the four alternative ribosomal proteins

## ***1.7. Mtb Average Expression Plots***

**Figure S5.** Illustration of the method for calculating and plotting the average expression of sets of genes. Mock data from two conditions (day 11 and day 19 of LDA infection), each with three samples, is used for demonstration. For simplicity, a gene set of three genes is used. The variance-stabilized data for each gene (batch adjusted using removeBatchEffect() function from the limma package in R) is first scaled and centered around zero to ensure comparability. Then, the expression values are averaged for each sample and adjusted so that zero represents the average of the untreated samples. Finally, these values are plotted, providing a comparison of gene expression between conditions for the gene sets of interest and illustrating the spread of gene expression across samples.

# **2. Supplemental Results**

## ***2.1 Host gene clustering***

Host genes were clustered into 8 groups, each with varying number of genes (**Fig S6**).

**Fig S6.** Number of genes sorted into each cluster after filtering to retain the 18,354 most variant host genes.

## ***2.2 Gene set enrichment of host gene clustering***

Gene set enrichment analysis was performed on each cluster of host genes (**Fig S7**) as described in the Mouse Pathway Enrichment Analysis section.

**Fig S7.** Dot plots illustrating the top 10 gene set enrichments for each cluster of host genes. Dot size represents adjusted p-value by Benjamini–Hochberg correction.

## ***2.3. PCA of Host samples***

To complement clustering, we carried out principal component analysis (PCA) on normalized counts for all detected genes. As with clustering, PCA revealed minimal separation of days 0, 7, and 11 samples, followed by a dramatic shift from day 11 to 19 (**Fig 2C**). However, whereas clustering implied static expression beyond day 19, PCA showed a modest but progressive drift of day 28 and 56 samples, indicating continued transcriptional remodeling during chronic infection. Moreover, PCA emphasized tight within‐timepoint cohesion of biological replicates, underscoring assay consistency (**Fig S8**).

**Fig S8.** PCA plot of mouse transcription at various stages of LDA infection. Each dot represents one mouse sample.

## ***2.4. Host pair-wise comparison of transcriptome between timepoints***

For each pair of time points available for LDA host RNA-seq data, the number of differentially expressed genes (DEGs) between the two time points was calculated as described in Differential Gene Expression of the Methods section. Most transcriptional changes occur between days 11 and 19 after infection. The number of DEGs then diminished after day 19 when infection reached the chronic stage in LDA infection (**Fig S9**).

**Fig S9.** Matrix of differentially expressed host genes between time points of LDA infection. Columns indicate reference and rows indicate comparison group. The upper right quadrant presents the total number of DEGs between time points. The lower left quadrant displays volcano plots where downregulated (blue) and upregulated (red) genes (circles) are plotted by the log fold change and adjusted p-value.

## ***2.5. Expression changes during late infection***

Although the host transcriptional profile appeared relatively stable throughout the chronic phase of infection, we observed a notable increase in the number of significant DEGs between days 28 and 56 when compared to the day 0 untreated control (**Fig 3C**). To better understand this shift, we asked two key questions: (1) were the same sets of genes consistently upregulated or downregulated during this period, and (2) what accounted for the rise in DEGs between days 28 and 56? Specifically, we aimed to determine whether the newly significant DEGs had previously shown no differential expression or whether some genes had switched directionality—shifting from significant upregulation to downregulation, or vice versa.

**Fig S10.** Alluvial plot showing host genes differentially expressed at days 19, 28, and 56 post-infection, relative to the day 0 untreated control. Genes not significantly differentially expressed at any time point were excluded from the analysis. Genes are colored by their expression pattern and directionality: dark red and dark blue indicate genes consistently upregulated or downregulated, respectively, across all three time points. Genes that alternated between significant upregulation and no differential expression are shown in light red, while those that alternated between significant downregulation and no differential expression are shown in light blue. Genes that switched direction—being significantly upregulated at one or more time points and significantly downregulated at others—are colored black.

Most genes that were significantly up- or downregulated on day 19 maintained the same direction of differential expression at days 28 and 56, indicating a largely stable transcriptional response during the chronic phase. Among the genes that did change over time, nearly all fluctuated between being significantly differentially expressed and not significant, but did not switch directionality. The increase in the number of differentially expressed genes observed by day 56 was largely driven by genes that had not previously reached significance, rather than by changes in expression direction. Only two genes reversed direction entirely—compared to day 0, both were significantly downregulated on day 19, not significantly different on day 28, and significantly upregulated by day 56 (**Fig S10**). Together, these findings suggest that during the chronic phase, the host transcriptome continued to diverge from the baseline (day 0), not by returning to a homeostatic or earlier state, but by amplifying existing expression patterns established earlier in infection.

## **2.6. Table S2.** *Expression changes of select host immune genes*

Genes canonically associated with host adaptive immunity significantly changed between days 11 and 19 of *Mtb* infection.

| **Gene** | **Log Fold Change** | **Adjusted p-value** | **Annotation** |
| --- | --- | --- | --- |
| Arg1 | -0.389967 | 0.32994887 | Arginase 1, liver |
| Arg2 | 1.48825437 | 1.20E-32 | Arginase type II |
| Cd14 | 2.23969238 | 2.28E-77 | CD14 |
| Cd177 | 2.29263868 | 2.61E-11 | CD177 |
| Cd2 | 0.78869239 | 1.24E-11 | CD2 |
| Cd209a | -0.3851578 | 0.03722886 | C-type lectin /DC-SIGN/CD209 |
| Cd247 | 0.59701447 | 2.91E-09 | CD247 |
| Cd274 | 3.49239669 | 1.09E-145 | CD274 antigen PD-L1 (B7-H1 or CD274) |
| Cd300a | 1.44114801 | 6.75E-21 | CD300A antigen |
| Cd300lf | 1.99007037 | 7.79E-47 | CD300 antigen like family member F |
| Cd3d | 1.19815823 | 1.14E-13 | CD3 delta antigen |
| Cd3g | 1.55472332 | 3.95E-22 | CD3 gamma antigen, |
| Cd4 | 1.20943609 | 1.84E-20 | CD4 |
| Cd44 | 1.0881044 | 1.62E-39 | CD44 |
| Cd52 | 1.95500447 | 6.18E-59 | CD52 antigen |
| Cd53 | 1.47305554 | 6.16E-35 | CD53 antigen/membrane late endosomas |
| Cd68 | 1.74632316 | 3.87E-62 | CD68/macrosiali lysosomal glycoprotein |
| Cd8a | 1.80305982 | 2.17E-24 | Cd8 alpha |
| Cd8b1 | 1.38592208 | 2.70E-12 | CD8beta1 |
| Clec4a3 | 1.89593751 | 2.51E-51 | C-type lectin domain family 4, member a3 |
| Clec4e | 5.17991636 | 9.01E-113 | C-type lectin domain family 4, member e/MINCLE |
| Clec4n | 2.16511529 | 5.04E-38 | C-type lectin domain family 4, member n/DECTIN-2 |
| Clec5a | 2.86273645 | 2.68E-62 | C-type lectin domain family 5, member a/MDL-1 |
| Clec7a | 2.60220374 | 3.71E-57 | C-type lectin domain family 7, member a/DECTIN-1 |
| Fasl | 1.88236506 | 2.90E-19 | Fas ligand (TNF superfamily, member 6) |
| Fcer1g | 2.31271892 | 3.28E-91 | FceRI gamma |
| Fcgr1 | 2.99927566 | 2.85E-70 | Fc gamma RI (CD64) |
| Fcgr2b | 1.81028012 | 4.66E-69 | Fc gamma RIIB (CD32) |
| Fcgr3 | 1.95874624 | 1.29E-70 | Fc gamma RIII (CD16) |
| Gzma | 1.78706381 | 1.81E-28 | Granzyme A |
| Gzmb | 2.72488878 | 1.71E-34 | Granzyme B |
| Gzmk | 4.43670989 | 2.90E-26 | Granzyme K |
| Ifi205 | 1.88420113 | 3.89E-70 | Interferon activated gene 205 |
| Ifi27 | 0.44384928 | 9.65E-10 | Interferon gamma inducible protein 30 |
| Ifi30 | 1.184226 | 3.84E-72 | Interferon-induced protein 35 |
| Ifi35 | 0.60908537 | 9.58E-22 | Interferon-induced protein 44 |
| Ifi44 | 2.58922862 | 2.55E-65 | Interferon gamma inducible protein 47 |
| Ifi47 | 2.78624097 | 4.47E-162 | Interferon-induced protein with tetratricopeptide repeats 1 |
| Ifit1 | 2.21330988 | 1.21E-84 | Interferon-induced protein with tetratricopeptide repeats 2 |
| Ifit2 | 1.59171127 | 2.40E-45 | Interferon-induced protein with tetratricopeptide repeats 3 |
| Ifit3 | 1.71878271 | 1.08E-46 | Interferon induced transmembrane protein 3 |
| Ifitm3 | 0.94896842 | 4.03E-32 | Interferon-induced protein 44 |
| Ifng | 5.39979473 | 3.07E-61 | Interferon gamma |
| Igtp | 3.79807226 | 1.69E-140 | Interferon gamma induced GTPase |
| Iigp1 | 3.97329564 | 2.02E-199 | Interferon inducible GTPase 1 |
| Il17d | -0.3184887 | 0.03359775 |  |
| Il17rc | 0.04823416 | 0.59259055 |  |
| Il17rd | -0.483445 | 9.07E-10 |  |
| Il17re | -0.2271525 | 0.00093103 |  |
| Il7r | 1.01543037 | 9.04E-15 | CD127/Interleukin 7 receptor) |
| Irf1 | 1.71561349 | 4.07E-121 | Interferon regulatory factor 1 |
| Irf5 | 1.62111094 | 1.51E-79 | Interferon regulatory factor 5 |
| Irf7 | 2.70649451 | 6.71E-74 | Interferon regulatory factor 7 |
| Irf8 | 1.60431339 | 4.43E-105 | Interferon regulatory factor 8 |
| Itgal | 1.713187 | 1.34E-33 | CD11a/Integrin alpha L |
| Itgam | 1.80902047 | 4.07E-18 | CD11b/ Integrin alpha M |
| Itgax | 1.76331182 | 1.41E-95 | CD11c/ Integrin alpha X |
| Itgb2 | 1.93240583 | 1.60E-50 | CD18/Integrin beta2 |
| Laptm5 | 1.37802634 | 6.32E-46 | Lysosomal-associated protein transmembrane 5 |
| Ltb | 1.22814841 | 3.47E-20 | Lymphotoxin B |
| Ltf | 2.06706525 | 0.00010409 | Lactotransferrin |
| Ly6i | 5.5099408 | 8.68E-146 | Lymphocyte antigen 6 complex, locus I |
| Ly75 | 1.44990403 | 5.97E-39 | C-type lectin/ DEC-205 |
| Mrc1 | 0.46881141 | 5.93E-06 | CD206 Mannose receptor, C type 1 |
| Ncf1 | 1.82430001 | 5.49E-68 | Neutrophil cytosolic factor 1/p47phox |
| Ncf2 | 1.30305049 | 9.77E-33 | Neutrophil cytosolic factor 2/p67 phox |
| Ncf4 | 1.76739383 | 5.65E-45 | Neutrophil cytosolic factor 4/p40phox |
| Nos2 | 3.53869251 | 1.04E-158 | Nitric oxide synthase 2, inducible, macrophage |
| Ptprc | 1.44280556 | 2.24E-34 | CD45 |
| Rab10 | 0.15816144 | 0.00334047 | RAB GTPase activating protein 1-like |
| Rab20 | 1.26867385 | 2.95E-33 | RAB geranylgeranyl transferase, b subunit |
| Rab24 | 0.1059952 | 0.13507396 | RAB guanine nucleotide exchange factor 1 |
| Rab32 | 1.7047507 | 1.02E-56 | Rab geranylgeranyl transferase, a subunit |
| Rab8a | 0.34251308 | 4.07E-11 | RAB guanine nucleotide exchange factor 1 |
| Slamf6 | 0.88749547 | 2.02E-10 | CD150 (SLAM family member 6) |
| Slamf7 | 2.46392789 | 1.75E-102 | CD319 (SLAM family member 7) |
| Slamf8 | 3.93518943 | 1.70E-145 | CD150 (SLAM family member 8) |
| Stat1 | 2.72877028 | 2.75E-142 | Signal transducer and activator of transcription 1 |
| Stat2 | 1.61996165 | 9.31E-110 | Signal transducer and activator of transcription 2 |
| Tlr1 | 1.59508875 | 1.74E-45 | Toll-like receptor 1 |
| Tlr12 | 0.44182793 | 0.00182068 | Toll-like receptor 12 |
| Tlr13 | 2.05931101 | 2.15E-43 | Toll-like receptor 13 |
| Tlr2 | 2.14629516 | 7.27E-68 | Toll-like receptor 2 |
| Tlr4 | 0.43552571 | 2.73E-08 | Toll-like receptor 4 |
| Tnf | 4.00475105 | 4.66E-100 | TNF-alpha |
| Tnfaip2 | 3.13545629 | 3.61E-112 | TNF-alpha-induced protein 2 |
| Tnfrsf1b | 1.79180147 | 3.14E-69 | TNFreceptor superfamily, member 1b |
| Traf1 | 0.41300551 | 4.38E-13 | Tnf receptor-associated factor 1 |
| Traf3 | 0.55669781 | 6.34E-19 | Tnf receptor-associated factor 3 |

## ***2.7. Bacterial gene clustering***

*Mtb* genes were clustered into 6 groups, each with varying number of genes (**Fig S11**).

**Fig S11.** Number of genes sorted into each cluster after filtering to retain the 3,317 variable *Mtb* genes.

## ***2.8. Table S3.*** *Gene categories that were significantly enriched in each gene cluster*

After clustering of bacterial genes, gene set enrichment analysis was run on each cluster. No gene categories were enriched for in clusters 2, 4, or 5.

| **cluster** | **Bacterial Functional Gene Category** | **Significant Genes** | **Set Size** | **Adjusted p-value** |
| --- | --- | --- | --- | --- |
| Cluster 1 | Carbohydrates organic acids and alcohols | 14 | 19 | 0.006 |
| Cluster 3 | DosR | 19 | 46 | 0.025 |
|  | Enduring Hypoxic Response | 42 | 138 | 0.032 |
|  | ESX3 | 6 | 7 | 0.025 |
| Cluster 6 | aerobic | 19 | 27 | 7.30E-05 |
|  | Amino acid biosynthesis | 41 | 78 | 4.30E-05 |
|  | Arabinogalactan (AG) | 9 | 15 | 0.046 |
|  | Aspartate family | 14 | 20 | 0.00083 |
|  | ATP-proton motive force | 8 | 8 | 4.00E-04 |
|  | Beta Oxidation | 11 | 18 | 0.018 |
|  | Cell wall synthesis | 24 | 36 | 2.40E-05 |
|  | Central intermediary metabolism | 21 | 38 | 0.002 |
|  | Cholesterol A and B ring degradation | 9 | 10 | 0.00064 |
|  | Drug targets | 16 | 33 | 0.046 |
|  | Energy Metabolism | 95 | 245 | 0.00061 |
|  | ESX1 | 12 | 17 | 0.002 |
|  | Fatty Acid Synthases II | 8 | 8 | 4.00E-04 |
|  | Glycolysis | 6 | 8 | 0.043 |
|  | KstR1 regulon | 33 | 64 | 0.00042 |
|  | Lipid Biosynthesis | 28 | 55 | 0.0016 |
|  | NADH dehydrogenase type I | 9 | 11 | 0.002 |
|  | PDIM | 17 | 20 | 4.50E-06 |
|  | Primary ribosomal proteins | 44 | 49 | 7.50E-18 |
|  | Protein and peptide secretion | 11 | 13 | 4.00E-04 |
|  | Ribosomal protein synthesis and modification | 44 | 53 | 2.60E-15 |
|  | Stringent Response - Repressed | 47 | 62 | 1.10E-13 |
|  | Synthesis and modification of macromolecules | 84 | 190 | 9.70E-06 |
|  | TCA cycle | 11 | 18 | 0.018 |

## ***2.9. Additional average expression plots of gene categories in LDA model***

**Fig S12.** Average gene expression of specified gene set categories in the LDA model. Each dot represents one murine sample.

## ***2.10. Expression changes in Sigma Factor genes***

**Fig S13.** The heatmap shows the batch-adjusted, VST-normalized, scaled gene expression averaged across timepoints for the LDA samples for the *Mtb* sigma factor genes.

The following table shows the log fold change (logFC) of gene encoding sigma factors on day 19, 28, and 56 compared to day 11 in the LDA model. The p-values were adjusted using the Benjamini–Hochberg^41^ method.

***Table S4.*** *LogFC of Sigma Factor genes*

| **gene** | **rv#** | **LDA D19 vs D11** | | **LDA D28 vs D11** | | **LDA D56 vs D11** | |
| --- | --- | --- | --- | --- | --- | --- | --- |
|  |  | **logFC** | **adj. p-value** | **logFC** | **adj. p-value** | **logFC** | **adj. p-value** |
| **sigA** | Rv2703 | -2.16 | <0.00001 | -2.06 | <0.00001 | -2.37 | <0.00001 |
| **sigB** | Rv2710 | 0.74 | 0.0739 | 1.18 | 0.00208 | 0.86 | 0.03235 |
| **sigC** | Rv2069 | -0.78 | 0.02588 | -0.4 | 0.25395 | -0.24 | 0.52909 |
| **sigD** | Rv3414c | -1.63 | <0.00001 | -2.03 | <0.00001 | -2.44 | <0.00001 |
| **sigE** | Rv1221 | 0.84 | 0.03697 | 1.92 | <0.00001 | 2.23 | <0.00001 |
| **sigF** | Rv3286c | 0.74 | 0.02323 | 0.88 | 0.00368 | 1.16 | 2.00E-04 |
| **sigG** | Rv0182c | 0.11 | 0.77763 | 0.2 | 0.54364 | -0.62 | 0.05432 |
| **sigH** | Rv3223c | 0.65 | 0.06377 | 0.97 | 0.00274 | 0.85 | 0.01233 |
| **sigI** | Rv1189 | 0.6 | 0.12139 | 0.62 | 0.09007 | 1.06 | 0.00404 |
| **sigK** | Rv0445c | 0.62 | 0.08444 | 0.07 | 0.85142 | 0.09 | 0.81951 |
| **sigL** | Rv0735 | 0.84 | 0.00659 | 0.13 | 0.68671 | 0.14 | 0.68005 |
| **sigM** | Rv3911 | 0.78 | 0.06977 | 0.78 | 0.05695 | 1.43 | 5.00E-04 |

## ***2.11. Expression changes in Toxin genes***

**Fig S14.** The heatmap shows the batch-adjusted, VST-normalized, scaled gene expression averaged across timepoints for the LDA samples for the *Mtb* toxin genes.

The following table shows the log fold change (logFC) of gene encoding toxins on day 19, 28, and 56 compared to day 11 in the LDA model. The p-values were adjusted using the Benjamini–Hochberg^41^ method.

***Table S5.*** *LogFC of Toxin genes*

| **gene** | **rv#** | **LDA D19 vs D11** | | **LDA D28 vs D11** | | **LDA D56 vs D11** | |
| --- | --- | --- | --- | --- | --- | --- | --- |
|  |  | **logFC** | **adj. p-value** | **logFC** | **adj. p-value** | **logFC** | **adj. p-value** |
| **Rv0059** | Rv0059 | -0.46 | 0.24235 | -0.79 | 0.02706 | -0.08 | 0.84434 |
| **Rv0299** | Rv0299 | -1.27 | 4.00E-05 | -1.15 | 0.00013 | -1.46 | 1.00E-05 |
| **Rv0836c** | Rv0836c | 0.92 | 0.11375 | 1.27 | 0.01857 | 1.49 | 0.00688 |
| **Rv0910** | Rv0910 | 0.28 | 0.32086 | 0.34 | 0.1864 | 0.59 | 0.02219 |
| **Rv0919** | Rv0919 | 0.46 | 0.27643 | 0.6 | 0.12093 | 0.89 | 0.02439 |
| **Rv1546** | Rv1546 | -0.55 | 0.07538 | -0.54 | 0.07012 | -0.42 | 0.19073 |
| **Rv1989c** | Rv1989c | -0.45 | 0.15834 | 0.66 | 0.02271 | 0.27 | 0.39483 |
| **Rv2019** | Rv2019 | 1.29 | 7.00E-05 | 1.41 | 1.00E-05 | 1.25 | 0.00015 |
| **Rv2022c** | Rv2022c | 0.57 | 0.09735 | 1.41 | <0.00001 | 0.96 | 0.00314 |
| **Rv2653c** | Rv2653c | 0.73 | 0.17714 | 1.09 | 0.02907 | 1.76 | 0.00051 |
| **Rv3180c** | Rv3180c | 0.27 | 0.52758 | 0.29 | 0.45347 | 1.09 | 0.00277 |
| **Rv3182** | Rv3182 | -0.31 | 0.36259 | -0.15 | 0.6467 | -0.1 | 0.77652 |
| **Rv3189** | Rv3189 | 0.51 | 0.14139 | 0.51 | 0.12123 | 1.16 | 0.00037 |
| **Rv3749c** | Rv3749c | 1.36 | <0.00001 | 1.12 | 1.00E-05 | 1.16 | 1.00E-05 |
| **dut** | Rv2697c | -0.14 | 0.73715 | 0.27 | 0.43416 | 0.32 | 0.38321 |
| **higB** | Rv1955 | 1.37 | 0.00012 | 2.28 | <0.00001 | 2.27 | <0.00001 |
| **mazF2** | Rv0659c | -0.5 | 0.18215 | -1.05 | 0.00153 | -1.56 | 1.00E-05 |
| **mazF3** | Rv1102c | -0.89 | 0.00697 | -1.13 | 0.00029 | -1.68 | <0.00001 |
| **mazF4** | Rv1495 | 0.67 | 0.03519 | 0.56 | 0.06435 | 0.71 | 0.02247 |
| **mazF5** | Rv1942c | -2.11 | <0.00001 | -1.76 | <0.00001 | -2.22 | <0.00001 |
| **mazF6** | Rv1991c | 0.94 | 0.00759 | 2.13 | <0.00001 | 2.23 | <0.00001 |
| **mazF7** | Rv2063A | -0.53 | 0.08465 | -0.79 | 0.0054 | -0.74 | 0.01474 |
| **mazF9** | Rv2801c | 0.6 | 0.06614 | 1.1 | 0.00019 | 0.73 | 0.02026 |
| **parE1** | Rv1959c | 0.28 | 0.42476 | 0.47 | 0.12182 | 0.91 | 0.00317 |
| **parE2** | Rv2142c | -0.01 | 0.98602 | 0.25 | 0.48043 | 0.45 | 0.20591 |
| **relE** | Rv1246c | 0.1 | 0.81476 | 0.39 | 0.24659 | -0.28 | 0.44738 |
| **relG** | Rv2866 | 0.9 | 0.01168 | 1.3 | 9.00E-05 | 1.32 | 0.00014 |
| **relK** | Rv3358 | 0.65 | 0.13667 | 0.53 | 0.20096 | 0.5 | 0.25087 |
| **vapC1** | Rv0065 | 0.51 | 0.13424 | 0.18 | 0.61285 | -0.04 | 0.91314 |
| **vapC10** | Rv1397c | -1.21 | 0.00188 | -1.15 | 0.00222 | -1.56 | 1.00E-04 |
| **vapC11** | Rv1561 | 0.36 | 0.37995 | 0.58 | 0.10488 | 1.11 | 0.0021 |
| **vapC12** | Rv1720c | 0.11 | 0.88884 | 0.27 | 0.67258 | 0.96 | 0.11828 |
| **vapC13** | Rv1838c | -0.05 | 0.92669 | -0.42 | 0.30362 | 0.75 | 0.06327 |
| **vapC14** | Rv1953 | -0.4 | 0.2231 | -0.16 | 0.62845 | -0.04 | 0.90121 |
| **vapC15** | Rv2010 | -0.9 | 0.01057 | -0.27 | 0.45818 | -0.57 | 0.11482 |
| **vapC16** | Rv2231A | 0.41 | 0.35123 | 0.62 | 0.11745 | 0.21 | 0.62987 |
| **vapC17** | Rv2527 | -0.08 | 0.81697 | -0.52 | 0.05874 | -0.36 | 0.22542 |
| **vapC18** | Rv2546 | 1.89 | 0.01483 | 2.78 | 0.00017 | 3.04 | 7.00E-05 |
| **vapC19** | Rv2548 | 0.1 | 0.78459 | 0.69 | 0.02057 | 0.91 | 0.00287 |
| **vapC2** | Rv0301 | -0.67 | 0.03232 | -0.88 | 0.00258 | -0.95 | 0.00207 |
| **vapC20** | Rv2549c | 0.19 | 0.63889 | 0.68 | 0.03607 | 1.11 | 0.00086 |
| **vapC21** | Rv2757c | 0.66 | 0.03697 | 0.74 | 0.01263 | 0.65 | 0.03754 |
| **vapC22** | Rv2829c | -0.23 | 0.49587 | -0.43 | 0.1467 | -0.41 | 0.1915 |
| **vapC23** | Rv2863 | 0.43 | 0.28845 | 0.71 | 0.05215 | 0.51 | 0.1915 |
| **vapC24** | Rv0240 | 0.09 | 0.80996 | 0.35 | 0.23756 | 0.31 | 0.33107 |
| **vapC26** | Rv0582 | 0.42 | 0.27145 | -0.14 | 0.72155 | -0.78 | 0.03389 |
| **vapC27** | Rv0598c | -0.87 | 0.00484 | -1.43 | <0.00001 | -1.34 | 2.00E-05 |
| **vapC28** | Rv0609 | -0.67 | 0.03971 | -1.15 | 0.00013 | -1.97 | <0.00001 |
| **vapC29** | Rv0617 | -1.12 | 0.00021 | -0.78 | 0.00962 | -1.6 | <0.00001 |
| **vapC3** | Rv0549c | 0.03 | 0.94748 | 0.71 | 0.01721 | 0.54 | 0.0822 |
| **vapC30** | Rv0624 | -0.08 | 0.82671 | -0.17 | 0.60129 | 0.53 | 0.09108 |
| **vapC31** | Rv0749 | -0.19 | 0.57612 | -0.11 | 0.72787 | 0.36 | 0.23872 |
| **vapC32** | Rv1114 | 0.38 | 0.19768 | -0.19 | 0.51102 | 0.36 | 0.21978 |
| **vapC33** | Rv1242 | -0.63 | 0.16769 | 0.03 | 0.94599 | -0.19 | 0.69733 |
| **vapC34** | Rv1741 | 0.31 | 0.39758 | 0.76 | 0.01688 | 1.16 | 0.00032 |
| **vapC35** | Rv1962c | -0.15 | 0.71267 | -0.05 | 0.88455 | -0.04 | 0.91922 |
| **vapC36** | Rv1982c | -1.37 | 1.00E-05 | -1.93 | <0.00001 | -2.2 | <0.00001 |
| **vapC37** | Rv2103c | -0.67 | 0.05119 | -0.4 | 0.23756 | -0.46 | 0.19546 |
| **vapC38** | Rv2494 | -0.39 | 0.28066 | -0.41 | 0.20894 | -0.48 | 0.15892 |
| **vapC39** | Rv2530c | 0.01 | 0.98035 | 0.06 | 0.83782 | -0.13 | 0.69906 |
| **vapC4** | Rv0595c | 0.1 | 0.77693 | 0.21 | 0.46946 | 0.33 | 0.25653 |
| **vapC40** | Rv2596 | 0.34 | 0.57663 | 0.5 | 0.35966 | 0.29 | 0.66421 |
| **vapC41** | Rv2602 | -0.68 | 0.03013 | -0.6 | 0.04417 | -0.84 | 0.00757 |
| **vapC42** | Rv2759c | 0.13 | 0.76526 | 0.46 | 0.18127 | 0.67 | 0.05743 |
| **vapC43** | Rv2872 | -0.49 | 0.1536 | -0.41 | 0.20117 | -0.44 | 0.19718 |
| **vapC44** | Rv3320c | -0.27 | 0.50985 | -0.37 | 0.30045 | -0.32 | 0.4105 |
| **vapC46** | Rv3384c | -0.3 | 0.42002 | -0.55 | 0.10305 | 0.42 | 0.24678 |
| **vapC47** | Rv3408 | 0.16 | 0.64715 | 0.13 | 0.67934 | -0.27 | 0.39993 |
| **vapC5** | Rv0627 | -0.08 | 0.85672 | 0.1 | 0.78938 | 0.56 | 0.10987 |
| **vapC6** | Rv0656c | 0.13 | 0.76993 | 0.43 | 0.228 | 0.92 | 0.00888 |
| **vapC7** | Rv0661c | 0.22 | 0.70099 | 0.45 | 0.35073 | 0.73 | 0.13652 |
| **vapC8** | Rv0665 | 0.11 | 0.79657 | -0.32 | 0.38526 | 0.28 | 0.46918 |
| **vapC9** | Rv0960 | -0.69 | 0.02751 | -0.63 | 0.0366 | -0.93 | 0.00304 |

## ***2.12. Concordance of host and Mtb transcription***

Mean normalized expression of select gene *Mtb* and host gene categories were plotted against each other. For each category, normalized expression values were first averaged across genes within the category for each mouse and then normalized relative to the mean expression of that category at day 11. The genes included within each Mtb and host gene category are given below.

***Table S6.*** *Genes in select host categories for host-pathogen analysis*

| **Category** | **Gene** |
| --- | --- |
| Activated T-cell Proliferation | Abl1, Ager, Arg1, Btn1a1, Btn2a2, Casp3, Cd24a, Cd274, Cd86, Crtam, Ephb6, Epo, Fadd, Fyn, Gpam, Hmgb1, Icosl, Ido1, Igf1, Igf2, Igfbp2, Il12b, Il12rb1, Il18, Il2, Il23a, Il2ra, Il4, Itgad, Itgal, Itgam, Itgax, Itgb2, Jak3, Laptm5, Lilrb4a, Lilrb4b, Lrrc32, Pdcd1lg2, Ppp3ca, Prkar1a, Prnp, Pycard, Rc3h1, Ripk3, Rps3, Satb1, Scrib, Slamf1, Stat5a, Stat5b, Tnfsf4, Tnfsf9 |
| Antigen Processing and Presentation | 2410137M14Rik, Ap3b1, Ap3d1, Arl8b, Atg5, B2m, Bag6, Calr, Ccl19, Ccl21a, Ccr7, Cd1d1, Cd1d2, Cd68, Cd74, Clec4b2, Ctse, Ctsl, Ctss, Ext1, Fcer1g, Fcer2a, Fcgr1, Fcgr2b, Fcgr3, Fcgr4, Fgl2, Flt3, Gba, Gm11127, Gm8909, H2-Aa, H2-Ab1, H2-Bl, H2-D1, H2-DMa, H2-DMb1, H2-DMb2, H2-Ea, H2-Eb1, H2-Eb2, H2-K1, H2-M1, H2-M10.1, H2-M10.2, H2-M10.3, H2-M10.4, H2-M10.5, H2-M10.6, H2-M11, H2-M2, H2-M3, H2-M5, H2-M9, H2-Oa, H2-Ob, H2-Q1, H2-Q10, H2-Q2, H2-Q4, H2-Q6, H2-Q7, H2-T-ps, H2-T10, H2-T22, H2-T23, H2-T24, H2-T3, H60b, H60c, Hfe, Icam1, Ide, Ifi30, Ifng, Ighe, Ighm, Iglc4, Kdm5d, Marchf1, Marchf8, Mfsd6, Mill1, Mill2, Mr1, Nod1, Nod2, Pdia3, Pikfyve, Psap, Psmb8, Psmb9, Psme1, Psme2, Ptpn22, Pycard, Rab10, Rab27a, Rab32, Rab33a, Rab34, Rab35, Rab3b, Rab3c, Rab4a, Rab5b, Rab6a, Rab8b, Raet1d, Raet1e, Relb, Rftn1, Slc11a1, Tap1, Tap2, Tapbp, Tapbpl, Thbs1, Traf6, Trem2, Treml4, Trex1, Ulbp1, Unc93b1, Was, Washc1, Wdfy4, Ythdf1 |
| B-cell Activation | 6030468B19Rik, Abl1, Ada, Adam17, Adgrg3, Ahr, Aicda, Akirin2, Ankle1, Aplf, Aqp8, Atad5, Atm, Atp11c, Bad, Bak1, Bank1, Batf, Bax, Bcl11a, Bcl2, Bcl3, Bcl6, Blnk, Bmi1, Bst1, Btk, Btla, Card11, Casp3, Ccr6, Cd180, Cd19, Cd22, Cd24a, Cd27, Cd28, Cd300a, Cd320, Cd38, Cd40, Cd40lg, Cd70, Cd74, Cd79a, Cd79b, Cd81, Cd86, Cdh17, Cdkn1a, Cdkn2a, Cebpg, Cfb, Chrna4, Chrna7, Chrnb2, Clcf1, Cmtm7, Cr2, Ctla4, Ctps, Cxcr5, Cyld, Dcaf1, Dclre1c, Dll1, Dnajb9, Dock10, Dock11, Dpp4, Enpp1, Ep300, Ephb2, Ercc1, Exo1, Exosc3, Exosc6, Ezh2, Fas, Fcgr2b, Fcrl1, Flt3, Fnip1, Fosl2, Foxj1, Foxp1, Foxp3, Fzd9, Gapt, Gimap1, Gm13271, Gm13272, Gm13275, Gm13276, Gm13277, Gm13283, Gon4l, Gpr183, Gps2, H2-Ab1, Hdac5, Hdac7, Hdac9, Hhex, Hmces, Hmga1, Hmgb3, Hspd1, Icosl, Id2, Ifna1, Ifna11, Ifna12, Ifna13, Ifna14, Ifna15, Ifna16, Ifna2, Ifna4, Ifna5, Ifna6, Ifna7, Ifna9, Ifnab, Ifnb1, Ifne, Ifng, Ifnk, Ifnz, Igbp1, Igbp1b, Ighd, Ighe, Ighg1, Ighm, Igkc, Igkj5, Ikzf1, Ikzf3, Il10, Il13, Il2, Il21, Il27ra, Il2rg, Il3, Il4, Il4i1, Il5, Il6, Il7, Il7r, Il9, Il9r, Inpp5d, Irf2bp2, Irf8, Irs2, Itfg2, Itm2a, Jak3, Kit, Kmt5b, Kmt5c, Laptm5, Lat2, Lax1, Lef1, Lfng, Lgals1, Lgals8, Lig4, Lilrb4a, Lyl1, Lyn, Mad2l2, Malt1, Mef2c, Mfng, Mif, Mir150, Mir17, Mir18, Mir181b-1, Mir181b-2, Mir19a, Mir19b-1, Mir20a, Mir92-1, Mlh1, Mmp14, Ms4a1, Msh2, Msh6, Myb, Mzb1, Nbn, Nckap1l, Ndfip1, Nfam1, Nfatc1, Nfatc2, Nfkbiz, Nhej1, Nkx2-3, Nod2, Notch2, Nsd2, Ntrk1, Onecut1, Pagr1a, Parp3, Pawr, Paxip1, Pcid2, Peli1, Pfdn1, Phb, Phb2, Phf14, Pik3cd, Pik3r1, Pkn1, Plcg2, Plcl2, Pms2, Pnp, Polm, Pou1f1, Pou2af1, Pou2f2, Ppp2r3c, Prkcb, Prkcd, Prkdc, Prlr, Pten, Ptk2b, Ptpn2, Ptpn6, Ptprc, Ptprj, Rabl3, Rag1, Rag2, Rasgrp1, Rbpj, Rc3h1, Rif1, Rnf168, Rnf8, Samsn1, Sanbr, Sash3, Sfrp1, Sh3kbp1, Shb, Shld1, Shld2, Shld3, Siglecg, Skap2, Slamf8, Slc15a4, Slc25a5, Slc39a10, Slc39a7, Sp3, Spi1, Spib, St3gal1, Stat5a, Stat5b, Stat6, Supt6, Swap70, Syk, Syvn1, Tbc1d10c, Tbx21, Tcf3, Tcirg1, Tfrc, Tgfb1, Themis2, Thoc1, Ticam1, Tirap, Tlr4, Tlr9, Tnfaip3, Tnfrsf13b, Tnfrsf13c, Tnfrsf21, Tnfrsf4, Tnfsf13, Tnfsf13b, Tnfsf4, Tnip2, Top2b, Tpd52, Traf3ip2, Trp53, Trp53bp1, Tsc2, Tshr, Txlna, Tyrobp, Ung, Vav3, Vpreb1a, Vpreb1b, Wnt3a, Xbp1, Xrcc4, Yy1, Zbtb1, Zbtb7a, Zfp36l1, Zfp36l2 |
| Macrophage Activation | Adgrf5, Ager, Aif1, App, Atm, Bpi, C1qa, C5ar1, Casp1, Cd1d1, Cd200, Cd84, Cebpa, Clu, Crtc3, Csf1r, Cst7, Ctsc, Cx3cl1, Cx3cr1, Dysf, Edn2, Fcer2a, Fer1l5, Foxp1, Gpr137b, Grn, Hamp, Havcr2, Hmgb1, Hspa4, Hspd1, Ifi35, Ifnb1, Ifng, Ifngr1, Ifngr2, Ighe, Il10, Il13, Il1rl1, Il33, Il4, Il4ra, Irgm1, Itgam, Jak2, Jmjd6, Jun, Jund, Kars, Kcnj8, Lbp, Ldlr, Lgals9, Lrfn5, Lrrk2, Mfhas1, Mir505, Mir7116, Mmp8, Muc5b, Myo18a, Naglu, Nampt, Nmi, Nr1d1, Nr1h3, Pja2, Pla2g10, Pla2g3, Pla2g4a, Pla2g5, Plcg2, Pparg, Prkce, Raet1d, Rora, Sbno2, Shpk, Slc11a1, Slc7a2, Snca, Snhg20, Sphk1, Stap1, Stk39, Sucnr1, Syk, Syt11, Tafa3, Tff2, Thbs1, Ticam1, Tlr1, Tlr2, Tlr3, Tlr4, Tlr6, Tlr9, Tmem106a, Tmem229b, Tnf, Tnip2, Trem2, Trex1, Trpv1, Ttbk1, Tyrobp, Ulbp1, Wnk4, Wnt5a |
| Neutrophil Activation | Abr, Anxa1, Anxa3, Bcr, Camp, Ccl5, Cd177, Cd300a, Ctsg, Cxcr2, Dnase1, Dnase1l3, F2rl1, Fcer1g, Fcgr4, Grn, Il15, Il16, Il18, Il18rap, Itgam, Itgb2, Itgb2l, Kmt2e, Lypd10, Lypd11, Myd88, Myo1f, Pikfyve, Pla2g2a, Plpp6, Pram1, Prg3, Prkcd, Ptafr, Scnn1b, Spi1, Stx11, Syk, Tnf, Traf3ip2, Tyrobp |

***Table S7.*** *Genes in select Mtb categories for host-pathogen analysis*

| **Category** | **Gene** |
| --- | --- |
| DosR Regulon | Rv1812c, Rv3126c, Rv0571c, Rv3132c, Rv2007c, Rv2032, Rv3127, Rv2029c, Rv0080, Rv1733c, Rv2624c, Rv1736c, Rv2030c, Rv3133c, Rv2003c, Rv2623, Rv0083, Rv0569, Rv3130c, Rv2028c, Rv1737c, Rv0570, Rv3128c, Rv3131, Rv0079, Rv0082, Rv3129, Rv3134c, Rv2627c, Rv2031c, Rv1738, Rv2628, Rv2631, Rv2005c, Rv0572c, Rv2004c, Rv1997, Rv1734c, Rv2626c, Rv2830c, Rv2625c, Rv1996, Rv1813c, Rv0574c, Rv0081, Rv2630, Rv2629, Rv2006 |
| Mycobactin Biosynthesis | Rv2377c, Rv2378c, Rv2379c, Rv2380c, Rv2381c, Rv2382c, Rv2383c, Rv2384, Rv2385, Rv2386c |
| Aerobic Respiration | Rv0527, Rv0529, Rv0694, Rv1451, Rv1542c, Rv1854c, Rv1872c, Rv2193, Rv2194, Rv2195, Rv2196, Rv2200c, Rv2249c, Rv2470, Rv3043c, Rv3145, Rv3146, Rv3148, Rv3150, Rv3151, Rv3152, Rv3153, Rv3154, Rv3155, Rv3156, Rv3157, Rv3158, Rv3302c |
| Primary Ribosomal Proteins | Rv0053, Rv0055, Rv0056, Rv0640, Rv0641, Rv0651, Rv0682, Rv0683, Rv0700, Rv0701, Rv0702, Rv0703, Rv0704, Rv0705, Rv0706, Rv0707, Rv0709, Rv0710, Rv0714, Rv0715, Rv0716, Rv0717, Rv0718, Rv0719, Rv0720, Rv0722, Rv0723, Rv0995, Rv1015c, Rv1298, Rv1630, Rv1642, Rv1643, Rv2412, Rv2441c, Rv2442c, Rv2785c, Rv2890c, Rv2904c, Rv2909c, Rv3241c, Rv3420c, Rv3442c, Rv3443c, Rv3456c, Rv3458c, Rv3459c, Rv3460c, Rv3461c, Rv3924c |

## ***2.13. DGEA in HDA model***

Differential gene expression analysis was run on the samples in the HDA model (**Fig S15**).

**Fig S15.** Matrix of differentially expressed *Mtb* genes between time points of HDA infection. Columns indicate reference and rows indicate comparison group. The upper right quadrant presents the total number of DEGs between time points. The lower left quadrant displays volcano plots where downregulated (blue) and upregulated (red) genes (circles) are plotted by the log fold change and adjusted p-value.

## ***2.14. Gene categories enriched in genes differentially expressed between models***

***Table S8.*** *Genes in select Mtb categories for model comparison*

| **Category** | **Gene** |
| --- | --- |
| DosR Regulon | Rv1812c, Rv3126c, Rv0571c, Rv3132c, Rv2007c, Rv2032, Rv3127, Rv2029c, Rv0080, Rv1733c, Rv2624c, Rv1736c, Rv2030c, Rv3133c, Rv2003c, Rv2623, Rv0083, Rv0569, Rv3130c, Rv2028c, Rv1737c, Rv0570, Rv3128c, Rv3131, Rv0079, Rv0082, Rv3129, Rv3134c, Rv2627c, Rv2031c, Rv1738, Rv2628, Rv2631, Rv2005c, Rv0572c, Rv2004c, Rv1997, Rv1734c, Rv2626c, Rv2830c, Rv2625c, Rv1996, Rv1813c, Rv0574c, Rv0081, Rv2630, Rv2629, Rv2006 |
| Enduring Hypoxic Response | Rv0048c, Rv0084, Rv0116c, Rv0140, Rv0171, Rv0188, Rv0196, Rv0232, Rv0233, Rv0244c, Rv0251c, Rv0324, Rv0327c, Rv0328, Rv0347, Rv0384c, Rv0474, Rv0494, Rv0520, Rv0521, Rv0551c, Rv0563, Rv0575c, Rv0614, Rv0653c, Rv0654, Rv0687, Rv0726c, Rv0753c, Rv0754, Rv0757, Rv0766c, Rv0767c, Rv0792c, Rv0793, Rv0826, Rv0827c, Rv0847, Rv0967, Rv0986, Rv0989c, Rv0991c, Rv1048c, Rv1088, Rv1152, Rv1169c, Rv1221, Rv1255c, Rv1284, Rv1375, Rv1403c, Rv1405c, Rv1471, Rv1472, Rv1578c, Rv1623c, Rv1628c, Rv1669, Rv1733c, Rv1755c, Rv1760, Rv1766, Rv1806, Rv1874, Rv1875, Rv1888c, Rv1891, Rv1909c, Rv1926c, Rv1955, Rv1956, Rv1960c, Rv1963c, Rv1974, Rv1985c, Rv1990c, Rv1994c, Rv2012, Rv2022c, Rv2034, Rv2050, Rv2122c, Rv2123, Rv2185c, Rv2299c, Rv2324, Rv2359, Rv2374c, Rv2389c, Rv2390c, Rv2412, Rv2465c, Rv2466c, Rv2497c, Rv2498c, Rv2499c, Rv2500c, Rv2504c, Rv2517c, Rv2526, Rv2557, Rv2558, Rv2583c, Rv2617c, Rv2626c, Rv2627c, Rv2628, Rv2642, Rv2653c, Rv2657c, Rv2662, Rv2663, Rv2687c, Rv2688c, Rv2693c, Rv2694c, Rv2699c, Rv2745c, Rv2830c, Rv2876, Rv2913c, Rv2964, Rv3007c, Rv3016, Rv3066, Rv3161c, Rv3176c, Rv3188, Rv3197A, Rv3206c, Rv3221A, Rv3223c, Rv3290c, Rv3334, Rv3406, Rv3418c, Rv3480c, Rv3503c, Rv3515c, Rv3531c, Rv3537, Rv3571, Rv3574, Rv3681c, Rv3747, Rv3848, Rv3862c, Rv3864, Rv3913 |
| Oxidative Stress | Rv0324, Rv0353, Rv0823c, Rv1049, Rv1129c, Rv1674c, Rv1909c, Rv2374c, Rv2621c, Rv2710, Rv2745c, Rv2989, Rv3173c, Rv3223c, Rv3334, Rv3583c, Rv3840, Rv0338c, Rv0816c, Rv0848, Rv1285, Rv1286, Rv1336, Rv1461, Rv1462, Rv1463, Rv1464, Rv1465, Rv1466, Rv1471, Rv2377c, Rv2378c, Rv2379c, Rv2380c, Rv2381c, Rv2382c, Rv2383c, Rv2384, Rv2386c, Rv3841, Rv1908c, Rv3913, Rv3914, Rv1317c, Rv2737c, Rv3201c, Rv3202c, Rv3585 |
| Universal Stress Proteins | Rv1028c, Rv1636, Rv1996, Rv2005c, Rv2028c, Rv2319c, Rv2623, Rv2624c, Rv3134c |
| Mycobactin Biosynthesis | Rv2377c, Rv2378c, Rv2379c, Rv2380c, Rv2381c, Rv2382c, Rv2383c, Rv2384, Rv2385, Rv2386c |
| ESX-3 | Rv0282, Rv0283, Rv0284, Rv0287, Rv0288, Rv0289, Rv0290, Rv0291, Rv0292 |
| Zur Regulon | Rv0105c, Rv0106, Rv0280, Rv0281, Rv0282, Rv0283, Rv0284, Rv0287, Rv0288, Rv0289, Rv0290, Rv0291, Rv0292, Rv2055c, Rv2056c, Rv2057c, Rv2059 |
| Primary Ribosomal Proteins | Rv0053, Rv0055, Rv0056, Rv0640, Rv0641, Rv0651, Rv0682, Rv0683, Rv0700, Rv0701, Rv0702, Rv0703, Rv0704, Rv0705, Rv0706, Rv0707, Rv0709, Rv0710, Rv0714, Rv0715, Rv0716, Rv0717, Rv0718, Rv0719, Rv0720, Rv0722, Rv0723, Rv0995, Rv1015c, Rv1298, Rv1630, Rv1642, Rv1643, Rv2412, Rv2441c, Rv2442c, Rv2785c, Rv2890c, Rv2904c, Rv2909c, Rv3241c, Rv3420c, Rv3442c, Rv3443c, Rv3456c, Rv3458c, Rv3459c, Rv3460c, Rv3461c, Rv3924c |
| Synthesis and modification of macromolecules | Rv0001, Rv0002, Rv0003, Rv0005, Rv0006, Rv0009, Rv0041, Rv0053, Rv0054, Rv0055, Rv0056, Rv0058, Rv0071, Rv0105c, Rv0120c, Rv0126, Rv0182c, Rv0413, Rv0427c, Rv0429c, Rv0445c, Rv0629c, Rv0631c, Rv0639, Rv0640, Rv0641, Rv0651, Rv0667, Rv0668, Rv0682, Rv0683, Rv0684, Rv0685, Rv0700, Rv0701, Rv0702, Rv0703, Rv0704, Rv0705, Rv0706, Rv0707, Rv0709, Rv0710, Rv0714, Rv0715, Rv0716, Rv0717, Rv0718, Rv0719, Rv0720, Rv0722, Rv0723, Rv0735, Rv0861c, Rv0944, Rv0949, Rv0995, Rv1007c, Rv1010, Rv1015c, Rv1080c, Rv1189, Rv1210, Rv1221, Rv1253, Rv1292, Rv1297, Rv1298, Rv1299, Rv1316c, Rv1317c, Rv1326c, Rv1328, Rv1329c, Rv1364c, Rv1388, Rv1402, Rv1406, Rv1407, Rv1420, Rv1536, Rv1537, Rv1547, Rv1562c, Rv1563c, Rv1564c, Rv1629, Rv1630, Rv1633, Rv1638, Rv1640c, Rv1641, Rv1642, Rv1643, Rv1644, Rv1649, Rv1650, Rv1688, Rv1689, Rv1696, Rv1781c, Rv2055c, Rv2056c, Rv2057c, Rv2069, Rv2090, Rv2092c, Rv2130c, Rv2343c, Rv2357c, Rv2412, Rv2441c, Rv2442c, Rv2448c, Rv2464c, Rv2471, Rv2528c, Rv2533c, Rv2534c, Rv2555c, Rv2572c, Rv2580c, Rv2582, Rv2592c, Rv2593c, Rv2594c, Rv2614c, Rv2703, Rv2710, Rv2736c, Rv2737c, Rv2756c, Rv2783c, Rv2785c, Rv2793c, Rv2836c, Rv2838c, Rv2839c, Rv2841c, Rv2845c, Rv2882c, Rv2889c, Rv2890c, Rv2904c, Rv2906c, Rv2907c, Rv2909c, Rv2924c, Rv2973c, Rv2976c, Rv2985, Rv2986c, Rv2992c, Rv3009c, Rv3012c, Rv3014c, Rv3056, Rv3062, Rv3105c, Rv3198c, Rv3201c, Rv3202c, Rv3211, Rv3223c, Rv3232c, Rv3241c, Rv3263, Rv3286c, Rv3287c, Rv3296, Rv3297, Rv3336c, Rv3366, Rv3370c, Rv3414c, Rv3420c, Rv3442c, Rv3443c, Rv3455c, Rv3456c, Rv3457c, Rv3458c, Rv3459c, Rv3460c, Rv3461c, Rv3462c, Rv3580c, Rv3585, Rv3598c, Rv3644c, Rv3646c, Rv3649, Rv3674c, Rv3711c, Rv3715c, Rv3721c, Rv3731, Rv3834c, Rv3852, Rv3907c, Rv3911, Rv3924c |
| Protein translation and modification | Rv0009, Rv0120c, Rv0429c, Rv0684, Rv0685, Rv1080c, Rv1299, Rv1641, Rv2534c, Rv2582, Rv2839c, Rv2882c, Rv2889c, Rv3105c, Rv3462c |
| TCA cycle | Rv0066c, Rv0794c, Rv0889c, Rv0896, Rv0951, Rv0952, Rv1098c, Rv1131, Rv1240, Rv1248c, Rv1475c, Rv2215, Rv2498c, Rv2967c, Rv3316, Rv3317, Rv3318, Rv3319, Rv3339c |
| ATP Synthase | Rv1304, Rv1305, Rv1306, Rv1307, Rv1308, Rv1309, Rv1310, Rv1311 |

# **3. References**

1.	Musisi, E. *et al.* Reproducibility of the Ribosomal RNA Synthesis Ratio in Sputum and Association with Markers of Mycobacterium tuberculosis Burden. *Microbiol. Spectr.* **9**, (2021).

2.	Cole, S. T. *et al.* Deciphering the biology of Mycobacterium tuberculosis from the complete genome sequence. *Nature* **396**, 190–190 (1998).

3.	Lew, J. M., Kapopoulou, A., Jones, L. M. & Cole, S. T. TubercuList – 10 years after. *Tuberculosis* **91**, 1–7 (2011).

4.	Soni, D. K., Dubey, S. K. & Bhatnagar, R. ATP-binding cassette (ABC) import systems of Mycobacterium tuberculosis: target for drug and vaccine development. *Emerg. Microbes Infect.* **9**, 207–220 (2020).

5.	Kushwaha, A. K. & Bhushan, S. Unique structural features of the Mycobacterium ribosome. *Prog. Biophys. Mol. Biol.* **152**, 15–24 (2020).

6.	Karbalaei Zadeh Babaki, M., Soleimanpour, S. & Rezaee, S. A. Antigen 85 complex as a powerful Mycobacterium tuberculosis immunogene: Biology, immune-pathogenicity, applications in diagnosis, and vaccine design. *Microb. Pathog.* **112**, 20–29 (2017).

7.	Shao, Y. *et al.* TADB: a web-based resource for Type 2 toxin–antitoxin loci in bacteria and archaea. *Nucleic Acids Res.* **39**, D606–D611 (2011).

8.	Abrahams, K. A. & Besra, G. S. Synthesis and recycling of the mycobacterial cell envelope. *Curr. Opin. Microbiol.* **60**, 58–65 (2021).

9.	Schnappinger, D. *et al.* Transcriptional Adaptation of Mycobacterium tuberculosis within Macrophages: Insights into the Phagosomal Environment. *J. Exp. Med.* **198**, 693–704 (2003).

10.	Kirksey, M. A. *et al.* Spontaneous phthiocerol dimycocerosate-deficient variants of Mycobacterium tuberculosis are susceptible to gamma interferon-mediated immunity. *Infect. Immun.* **79**, 2829–2838 (2011).

11.	Pawełczyk, J. *et al.* Cholesterol-dependent transcriptome remodeling reveals new insight into the contribution of cholesterol to Mycobacterium tuberculosis pathogenesis. *Sci. Rep.* **11**, 12396 (2021).

12.	Tallman, K. R., Levine, S. R. & Beatty, K. E. Small-Molecule Probes Reveal Esterases with Persistent Activity in Dormant and Reactivating Mycobacterium tuberculosis. *ACS Infect. Dis.* **2**, 936–944 (2016).

13.	Lee, B. S., Sviriaeva, E. & Pethe, K. Targeting the cytochrome oxidases for drug development in mycobacteria. *Prog. Biophys. Mol. Biol.* **152**, 45–54 (2020).

14.	Nobre, A., Alarico, S., Maranha, A., Mendes, V. & Empadinhas, N. The molecular biology of mycobacterial trehalose in the quest for advanced tuberculosis therapies. *Microbiology* **160**, 1547–1570 (2014).

15.	Ditse, Z., Lamers, M. H. & Warner, D. F. DNA Replication in Mycobacterium tuberculosis. *Microbiol. Spectr.* **5**, (2017).

16.	Voskuil, M. I. *et al.* Inhibition of Respiration by Nitric Oxide Induces a *Mycobacterium tuberculosis* Dormancy Program. *J. Exp. Med.* **198**, 705–713 (2003).

17.	Remm, S., Earp, J. C., Dick, T., Dartois, V. & Seeger, M. A. Critical discussion on drug efflux in Mycobacterium tuberculosis. *FEMS Microbiol. Rev.* **46**, fuab050 (2021).

18.	Rustad, T. R., Harrell, M. I., Liao, R. & Sherman, D. R. The Enduring Hypoxic Response of Mycobacterium tuberculosis. *PLOS ONE* **3**, e1502 (2008).

19.	Gröschel, M. I., Sayes, F., Simeone, R., Majlessi, L. & Brosch, R. ESX secretion systems: mycobacterial evolution to counter host immunity. *Nat. Rev. Microbiol.* **14**, 677–691 (2016).

20.	Duan, X., Xiang, X. & Xie, J. Crucial components of mycobacterium type II fatty acid biosynthesis (Fas-II) and their inhibitors. *FEMS Microbiol. Lett.* **360**, 87–99 (2014).

21.	Slayden, R. A. & Barry, C. E. The role of KasA and KasB in the biosynthesis of meromycolic acids and isoniazid resistance in Mycobacterium tuberculosis. *Tuberc. Edinb. Scotl.* **82**, 149–160 (2002).

22.	Wipperman, M. F., Sampson, N. S. & Thomas, S. T. Pathogen roid rage: cholesterol utilization by Mycobacterium tuberculosis. *Crit. Rev. Biochem. Mol. Biol.* **49**, 269–293 (2014).

23.	Batt, S. M., Burke, C. E., Moorey, A. R. & Besra, G. S. Antibiotics and resistance: the two-sided coin of the mycobacterial cell wall. *Cell Surf. Amst. Neth.* **6**, 100044 (2020).

24.	Domenech, P., Reed, M. B. & Barry, C. E. Contribution of the Mycobacterium tuberculosis MmpL protein family to virulence and drug resistance. *Infect. Immun.* **73**, 3492–3501 (2005).

25.	Melly, G. & Purdy, G. E. MmpL Proteins in Physiology and Pathogenesis of M. tuberculosis. *Microorganisms* **7**, 70 (2019).

26.	Quadri, L. E. N., Sello, J., Keating, T. A., Weinreb, P. H. & Walsh, C. T. Identification of a *Mycobacterium tuberculosis* gene cluster encoding the biosynthetic enzymes for assembly of the virulence-conferring siderophore mycobactin. *Chem. Biol.* **5**, 631–645 (1998).

27.	Marrakchi, H., Lanéelle, M.-A. & Daffé, M. Mycolic Acids: Structures, Biosynthesis, and Beyond. *Chem. Biol.* **21**, 67–85 (2014).

28.	Cook, G. M., Hards, K., Vilchèze, C., Hartman, T. & Berney, M. Energetics of Respiration and Oxidative Phosphorylation in Mycobacteria. *Microbiol. Spectr.* **2**, (2014).

29.	Voskuil, M. I., Bartek, I., Visconti, K. & Schoolnik, G. K. The Response of Mycobacterium Tuberculosis to Reactive Oxygen and Nitrogen Species. *Front. Microbiol.* **2**, (2011).

30.	Rens, C., Chao, J. D., Sexton, D. L., Tocheva, E. I. & Av-Gay, Y. Roles for phthiocerol dimycocerosate lipids in Mycobacterium tuberculosis pathogenesis. *Microbiol. Read. Engl.* **167**, (2021).

31.	Maitra, A. *et al.* Cell wall peptidoglycan in Mycobacterium tuberculosis: An Achilles’ heel for the TB-causing pathogen. *FEMS Microbiol. Rev.* **43**, 548–575 (2019).

32.	Raynaud, C. *et al.* Phospholipases C are involved in the virulence of Mycobacterium tuberculosis. *Mol. Microbiol.* **45**, 203–217 (2002).

33.	Rohde, K. H., Abramovitch, R. B. & Russell, D. G. Mycobacterium tuberculosis invasion of macrophages: linking bacterial gene expression to environmental cues. *Cell Host Microbe* **2**, 352–364 (2007).

34.	Li, Y., Corro, J. H., Palmer, C. D. & Ojha, A. K. Progression from remodeling to hibernation of ribosomes in zinc-starved mycobacteria. *Proc. Natl. Acad. Sci.* **117**, 19528–19537 (2020).

35.	Dahl, J. L. *et al.* The role of RelMtb-mediated adaptation to stationary phase in long-term persistence of Mycobacterium tuberculosis in mice. *Proc. Natl. Acad. Sci. U. S. A.* **100**, 10026–10031 (2003).

36.	Hartman, T. *et al.* Succinate dehydrogenase is the regulator of respiration in Mycobacterium tuberculosis. *PLoS Pathog.* **10**, e1004510 (2014).

37.	Thanna, S. & Sucheck, S. J. Targeting the trehalose utilization pathways of Mycobacterium tuberculosis. *MedChemComm* **7**, 69–85 (2016).

38.	Maurya, R. K., Bharti, S. & Krishnan, M. Y. Triacylglycerols: Fuelling the Hibernating Mycobacterium tuberculosis. *Front. Cell. Infect. Microbiol.* **8**, 450 (2018).

39.	Wan, T. *et al.* Structural insights into the functional divergence of WhiB-like proteins in *Mycobacterium tuberculosis*. *Mol. Cell* **81**, 2887-2900.e5 (2021).

40.	Dow, A. *et al.* Zinc limitation triggers anticipatory adaptations in Mycobacterium tuberculosis. *PLoS Pathog.* **17**, e1009570 (2021).

41.	Benjamini, Y. & Hochberg, Y. Controlling the False Discovery Rate: A Practical and Powerful Approach to Multiple Testing. *J. R. Stat. Soc. Ser. B Methodol.* **57**, 289–300 (1995).
